# Delivery of an at-home transcranial direct current stimulation intervention to mitigate pain in patients with end-stage kidney disease receiving hemodialysis (ESKD/HD)

**DOI:** 10.3389/fpain.2023.1132625

**Published:** 2023-04-05

**Authors:** Jordan Van Zyl, Helena Knotkova, Patricia Kim, Charles R. Henderson, Russell K. Portenoy, Nathaniel Berman, Melissa W. Frederic, M. Carrington Reid

**Affiliations:** ^1^ MJHS Institute for Innovation in Palliative Care, New York, NY, United States; ^2^ Department of Family & Social Medicine, Albert Einstein College of Medicine, Bronx, NY, United States; ^3^ Division of Geriatrics and Palliative Medicine, Weill Cornell Medicine, New York, NY, United States; ^4^ Department of Psychology, Cornell University, Ithaca, NY, United States; ^5^ Department of Neurology, Albert Einstein College of Medicine, Bronx, NY, United States; ^6^ Rogosin Institute, Weill Cornell Medicine, New York, NY, United States; ^7^ Division of Nephrology and Hypertension, Weill Cornell Medicine, New York, NY, United States

**Keywords:** transcranial direct current stimulation (tDCS), non-invasive neurostimulation, end-stage kidney disease (ESKD), hemodialysis (HD), chronic pain, clinical trial protocol

## Abstract

**Background:**

Poorly controlled pain remains a problem for many patients with end-stage kidney disease requiring hemodialysis (ESKD/HD) and customary approaches to pain management (e.g., opioids, non-steroidals) confer substantial risk. Accordingly, non-pharmacologic therapies are needed for use in this population. Non-invasive transcranial Direct Current Simulation (tDCS) constitutes a promising nonpharmacologic method for pain management in affected individuals.

**Aims:**

This study seeks to: 1) determine the effects of an 8-week course of at-home tDCS vs. sham tDCS on pain intensity, pain interference, medication usage, quality of life, and mood; 2) determine if tDCS effects vary by race/ethnicity; and 3) ascertain patient satisfaction with device use.

**Methods:**

This double-blind, randomized, sham-controlled clinical trial will enroll 100 ESKD/HD patients with moderate-to-severe (≥4 on 0–10 scale) chronic pain. The active study intervention consists of 20 min of tDCS delivered over the primary motor cortex 5 days/week for 8 weeks. The comparator is a sham procedure that provides no effective stimulation. The primary outcome analysis will evaluate efficacy of tDCS for pain reduction after two months of stimulation. We will also assess the effects of treatment on analgesic consumption, pain interference, depressed mood, and quality of life. The statistical plan will include fixed classification factors for treatment (vs. sham), clinic sites, and assessment time, and the interaction of these factors adjusting for covariates (e.g., race/ethnicity, pain level).

**Conclusion:**

At-home tDCS constitutes a promising nonpharmacologic treatment for pain mitigation in persons with ESKD/HD. This unique RCT could transform the way pain is managed in this vulnerable population.

**Trial Registration:**

NCT05311956.

## Background

The prevalence of end-stage kidney disease requiring chronic hemodialysis (ESKD/HD) is rising and racial/ethnic minorities are disproportionately affected (1–3). More than 30% of ESKD/HD are Black patients, and during the past two decades, the prevalence of ESKD/HD has increased by more than 70% in the Hispanic population (4–6). Chronic pain is highly prevalent among those with ESKD/HD and pain intensity is associated with mortality, particularly among racial/ethnic minorities (7). Patients develop ESKD as a consequence of many different conditions, and suffer from disparate comorbidities; pain syndromes are therefore heterogeneous in this population, including pain stemming from neuropathic, musculoskeletal, orthopedic, and rheumatologic disorders, as well as the discomfort arising from ESKD and dialysis itself. Thus, many patients with ESKD present with mixed pain disorders that include nociceptive, neuropathic as well as nociplastic components. Conventional treatment for chronic pain relies on systemic drug therapy with opioid drugs or adjuvant analgesics, but evidence of efficacy is limited and the potential for drug-disease interactions confer significant risk of adverse outcomes (8–10). There is a compelling need for novel analgesic treatment approaches that pose less risk in this medically vulnerable population.

Transcranial direct current stimulation (tDCS) is a non-invasive neuromodulatory intervention, designated by the United States. Food and Drug Administration as having minimal risk, that may reduce pain and analgesic consumption in patients with diverse types of chronic pain (11–18). tDCS delivers low intensity (1 or 2 milliamperes) electrical current through the skull to selected areas of the brain and induces changes in excitability and activation of brain neurons and neuronal circuits. The primary mechanism of tDCS is a subthreshold modulation of neuronal resting membrane potential. Stimulation for a few minutes results in neuroplasticity of glutamatergic synapses that may be associated with longer-term effects (19–21). In addition, recent evidence suggests that tDCS interacts with various neurotransmitters in the brain, such as dopamine, acetylcholine, serotonin or GABA, and can also trigger changes in brain-derived neurotrophic factor (BDNF) that is associated with pain processing (22–25). These effects may up-regulate or down-regulate functional connectivity within brain networks, such as those important for pain processing (26).

The most common anatomical target for tDCS in pain management is the primary motor cortex (M1). Research suggests that the analgesic effects of M1 stimulation involve multiple neural circuits and are at least partially attributed to modulation of thalamic activity, motor-cortex-driven inhibition of the somatosensory cortex, and modulation of endogenous opioid release (27–29).

Numerous studies of various populations with acute pain as well as those with various chronic pain disorders conclude that tDCS can reduce pain and opioid consumption, and improve QoL, without the risk of serious adverse events. A recent meta-analysis of 27 randomized controlled trials (RCTs) using various tDCS stimulation protocols in patients with chronic pain demonstrated analgesic efficacy of tDCS as compared to sham, complemented by improved QoL (30). However, this evidence has important gaps. Previous RCTs, including an ongoing study of tDCS for pain in ESKD/HD (31), have been limited by small sample sizes and brief stimulation protocols, have not employed an at-home stimulation component, and assessed short-term outcomes only. To our knowledge, no tDCS analgesic trial has evaluated longer-term treatment effects or determined whether treatment effects vary by race/ethnicity.

RCTs of tDCS that include larger and heterogeneous samples and assess short-term and longer-term outcomes are needed to establish whether tDCS could transform the way pain is managed in the growing and ethnically diverse population of ESKD/HD patients. We are conducting a randomized trial evaluating 8-weeks of at-home tDCS in 100 adults with moderate-to-severe chronic pain due to ESKD/HD (score of ≥4 on a 0–10 scale). Double-blind assignment to either active stimulation or sham ensues for 8 treatment weeks. Change in pain intensity after 8 weeks is the primary outcome. Change in pain intensity after 2, 12, 16, and 26 weeks constitute secondary outcomes. Additional secondary outcomes include changes in analgesic drug use, pain interference, mood, and quality of life (Aim 1) after 8 and 26 weeks. The study also examines racial/ethnic differences in these tDCS effects (Aim 2) and ascertains the tolerability of tDCS and satisfaction with the device and procedure (Aim 3).

### Hypotheses

We hypothesize that the active tDCS stimulation applied over M1 for 20 min per day, 5 days per week for 8 weeks, at the intensity of 2 mA, will significantly reduce pain, lessen analgesic consumption, and improve QoL; and that the analgesic effects of tDCS will extend into the follow-up period. We also hypothesize that no significant treatment differences will be found across the three primary race/ethnicity groups targeted in this study and that tolerability of and satisfaction with the intervention will be high.

## Methods

### Study design

This trial employs a double-blind, sham controlled, randomized, 2-parallel arm design. ESKD/HD patients are screened for eligibility criteria at participating dialysis centers. Eligible patients who provide informed consent are stratified to ensure that roughly equal numbers of Hispanic or Latino(a), Black or African American, and non-Hispanic White participants, assigned to the active and sham treatment arms, respectively. Each patient undergoes 8 study visits over the 26-week study period, including consenting and screening (V1); tDCS familiarization/training (V2); baseline assessment and tDCS refresh training, device deployment and first tDCS application under supervision by study personnel (V3); outcome assessment at 2 weeks (V4); outcome assessment at the 8-week conclusion of the study intervention (V5); and finally, outcome assessments at 12 weeks (V6), 16 weeks (V7), and 26 weeks (V8) from baseline. This study has institutional review board approval from Ethical & Independent Review Services (22,048).

### Screening, recruitment, randomization

Potential participants are identified by staff at the dialysis centers. Staff ascertain patient interest and those who agree to be contacted by study personnel complete an “agree-to-contact” sheet. Study personnel discuss consent in person or remotely, reviewing each section of the consent in detail. The process is designed to allow enough time for the patient and family to obtain sufficient information about the study in the manner that is not overwhelming and answers all questions before the patient decides whether to participate and then sign the secure e-consent (DocuSign) form.

Patients who provide consent undergo confirmation of eligibility for the study (Table 1). Eligible patients receive training in the use of the tDCS device and undergo random assignment to active tDCS or sham. Random assignment is performed in double-blind fashion. Information about the patient is provided to an unblinded study team member who determines assignment from a computer-generated list. The unblinded team member provides the blinded study team member with the identification number of the device that will be delivered to the participant. At Visit 3, the patient completes baseline measures and the team member who is blinded to the programming of the tDCS device provides refresher training about stimulation procedures and observes the patient performing the first stimulation. The patient interacts with the blinded study team member throughout the 8-week period of daily treatments, and during the assessment period that follows. The tDCS device is returned after the 8 weeks of study treatments.

**Table 1:** Inclusion and exclusion criteria.

Inclusion Criteria	Exclusion Criteria
• Age 21 years or older • Diagnosis of end-stage kidney disease (ICD 18.6) • Receiving hemodialysis at a Rogosin site • Montreal Cognitive Assessment (MoCA-Blind) adjusted score >21 • Pain for ≥3 months, • Self-report pain intensity of ≥4 (on a 0–10 scale) for the preceding week • Speaks English • Medically stable, as determined by the treating clinician. Defined as *unlikely to undergo a substantial change in illness or treatment during the next 3 months* • Able to provide written informed consent	• Active medical or major psychiatric illnesses that will impact pain or interfere with study procedures • History of head trauma, seizures, brain surgery, stroke, or cancer affecting the head • Use of another neurostimulation device (such as spinal cord stimulator, cardio-stimulator implanted cardioverter-defibrillator) • Metal implants in the head • Compromised skin integrity on the head in the area where electrodes will be placed • Does not have pain for ≥3 months, • Does not have a self-reported pain intensity of ≥4 (on a 0–10 scale) • Adjusted MoCA-Blind Score ≤21 • Unable and/or unwilling to provide informed consent • Does not tolerate tDCS at a skin test (performed at training Visit 2)

### Study measures

A complete list of all variables obtained at baseline and follow-up visits is shown in Table 2 below.

**Table 2:** Study measures.

Measure	Variable	Instrument	Assessment						
			V1	B	W2	W8	W12	W16	W26
**Primary**	Worst pain Intensity over the past 7 days	PROMIS pain Intensity short form		x		x			
**Secondary**	Worst pain Intensity over the past 7 days	PROMIS pain Intensity short form		x	x	x	x	x	x
	Average pain intensity over the past 7 days	PROMIS pain Intensity short form		x	x	x	x	x	x
	Prescription and over the counter analgesic medication use	5-day look back of use to include number of days of use and total dose of each analgesic taken		x	x	x	x	x	x
	Pain interference	PROMIS pain Interference		x	x	x	x	x	x
	Depressed mood	PHQ-8 Depression Questionnaire		x	x	x	x	x	x
	Quality of Life	World Health Organization Quality of Life Questionnaire (WHOQOL-BREF)		x	x	x	x	x	x
	Satisfaction with device and associated procedures	tDCS user survey				x			
	Safety and tolerability	Side effects and adverse events		x	x	x	x	x	x
	Potential moderators	Gender, age, pain level, race/ethnicity status	x						
**Covariates**	Social support	Lubben Social Network		x					
	Pain duration	How long have you experienced chronic pain? (years/months)		x					
	Comorbidities/# of chronic conditions	Charlson Comorbidity Index		x					
	Treatment credibility and expectation	Expectation for Treatment Scale		x		x			
	Functional status	OARS—Functional		x		x			x
	Current non-pharmacological methods of pain management	Participants’ answer open-ended questions on methods of managing pain and frequency of these methods		x		x			x
	Physical activity	PASE Scale		x		x			x
	Anxiety level	GAD-7		x	x	x	x	x	x
	Fatigue	PROMIS Fatigue Short Form 34a		x	x	x	x	x	x
	Neuropathic pain	PROMIS Neuropathic Pain		x	x	x	x	x	x
	Intervention Fidelity	Number of complete, incomplete, and missed sessions		x	x	x			
	Evaluation of blinding	Participants’ answer to the question of what modality (sham or active tDCS) they received				x			
	Dialysis symptom burden	Dialysis Symptom Index				x			
	User satisfaction	tDCS User Satisfaction Survey				x			

### Primary and secondary outcomes

The primary outcome for Aim 1 is change in worst pain intensity over the past seven days measured by the PROMIS pain Intensity short form after 8 weeks of the study intervention. Secondary outcomes include change in worst pain intensity after 2, 8, 12, 16, and 26 weeks after baseline as well as analgesic consumption, pain interference with function, depressed mood, and QoL. In addition, we are assessing the tolerability of the treatment in terms of side effects and adverse events reported through the study. Quality of blinding is being assessed by participants' guess at the end of the 8-week intervention of what treatment modality (sham or active tDCS) they received, and fidelity to the treatment is evaluated by the number of incomplete stimulation sessions during the 8-week intervention.

### The tDCS intervention

Participants randomized to the active tDCS group receive 20 min of direct current at the intensity of 2 mA once a day 5 days/week for 8 weeks, delivered *via* 2 sponge electrodes of size 5 × 5 cm presoaked by the manufacturer with normal saline and placed on the head using an EasyStrap headband for accurate electrode placement. The electrodes are inserted into a size-fitted headgear that allows for accurate and replicable positioning of the electrodes by patients at home (32). In the case of unilateral pain, the electrodes are placed with the anode over M1 contralateral to the pain-affected side of the body, and the cathode is placed over the supraorbital region on the hemisphere contralateral to the anode placement. In case of bilateral pain, the anode is placed over M1 of the left hemisphere and the cathode over the supraorbital region on the right. Devices programmed to sham produce 1 min of direct current that is ramped up to 2 mA over 30 s, ramped down over 30 s, and stay at 0 current for the remaining time. This model of sham mimics the sensory sensation of real stimulation without inducing neuroplasticity changes and has been successfully employed in numerous tDCS studies (33–35).

The tDCS device used to deliver the study intervention is a Soterix Mini-CT (Soterix Medical Inc.), programmed either to active tDCS or sham. The device has built-in dose control only allowing the user to apply the pre-determined dose each day, suitable for use at home. The device is paired with a tablet equipped with cellular connection, which enables video connection providing a real-time linkage to study personnel who can supervise and provide remote assistance. Participants do not need to have Internet connection at home to participate.

Participants and caregivers are trained in the use of the device at study Visit 2 and Visit 3. The first treatment using the device programmed to either active tDCS or sham is performed under the observation of the study team member. Subsequently, the team member contacts the patient prior to each application to provide the electronic code that unlocks the daily stimulation dose. This ensures that the device is being used correctly in compliance with the protocol, and allows for tracking of any adverse events. Refamiliarization on tDCS equipment is offered as needed.

To promote adherence and retention, patients are assigned to one staff member for the intervention and receive reminders prior to all study visits. They also receive materials on tDCS usage. Co-participation of each participant's informal caregiver is encouraged. Participants who do not have access to an informal caregiver and decide to participate alone are helped *via* video-connection and technical-assistance visits by the field study personnel. Patients who participate in the trial receive compensation for their time.

### Statistical plan

The analysis evaluates outcomes using models that adjust for covariates, including race/ethnicity and other patient characteristics such as gender, age, body mass index, cognitive status, and baseline pain level. The core model for evaluation of the tDCS intervention includes fixed classification factors for treatment (active vs. sham), clinic sites, and time of assessment (baseline, and 2, 8, 12, 16, and 26 weeks after baseline, giving us 6 assessment points for each participant); the interaction of these factors; and individuals as levels of a random classification factor. All variables as described in Table 1 will be examined for inclusion in the evaluation models, specifically race/ethnicity and all additional health and sociodemographic patient variables (e.g., age, education, gender, mental health, time on hemodialysis, pain level reported, PHQ-8 score, and comorbid conditions) as classification factors or covariates. The primary and secondary outcomes will be analyzed in the same core model.

There will be a focus on interactions of the other independent variables with treatment and time. Interactions are examined under Aim 1 as to obtain correctly specified models; potential moderators are examined in greater detail in the analysis for Aim 2 to determine whether treatment effects hold only for or are stronger for certain model subgroups or for a certain time points to determine anticipated reduction in treatment effects over time. Analysis for Aim 3, examining the tolerability and patient satisfaction with device and procedure will be examined in models of the same type as for Aims 1 and 2 to estimate levels of satisfaction and tolerability overall and whether these differ by factors such as race/ethnicity, gender, age, and pain level. Using data collected during each of the total of 40 stimulation sessions, we will examine the relationship between completed/successful sessions and better outcomes, i.e., if higher number of completed sessions results in better pain relief.

Finally, we will undertake a responder analysis to determine the proportion of participants in each group that achieve a pre-defined level of improvement in their pain levels. We consider a change score of −2.0 on our primary outcome measure or a percent change of >30% on our primary outcome measure relative to baseline to constitute a clinically meaningful change. We will also conduct analyses to assess whether treatment effects vary as a function of pain site with respect to our primary outcome (pain reduction) and our secondary outcomes including quality of life and analgesic consumption.

### Sample size

To achieve the planned sample size of 100 intent-to-treat participants, we are identifying through prescreening activities as many as 500 patients to produce 125 consenting and potentially eligible patients, i.e., endorse the presence of a pain problem and speak English. We estimate that approximately 80% of the 125 patients who pass the initial screen will be found eligible to participate after undergoing the full screening assessment, yielding 100 consented participants that will be randomized to active (or sham) treatment. With an estimated attrition rate of 20%, we are conducting follow-up assessments on approximately 80 patients at the scheduled 26-week assessment, with greater numbers at earlier assessments. Participants are recruited by race/ethnicity status to ensure roughly equal numbers of non-Hispanic White, Black or African American, and Hispanic or Latino(a) participants are enrolled. No participant is excluded based on race/ethnicity.

Power calculations for main outcome variables are given under the assumptions of a Type I error of.05, a Type II error of.20 (power of.80), 10 percent of the variance accounted for by other fixed terms in the model, a ratio of patient variance to error variance of 1.3, and a sample of 95 patients at the 8-week assessment. Based on means and standard deviations from previous studies for the primary and secondary outcome variables, detectable effect sizes for each variable are computed in a mixed model as described in the preceding section. Table 3 shows for selected outcomes the detectable treatment mean difference (the smallest detectable change resulting from the intervention) for treatment differences (baseline to 8 weeks) and for differences limited to a single level of a second variable such as race/ethnicity. With the target *n* = 100, we have adequate power to detect clinically meaningful differences for each of the main outcomes for 2-way interactions.

**Table 3:** Power calculations.

Outcome	Baseline Mean	Detectable Mean Treatment Difference	Detectable Difference for a Single Ethnicity Group
Pain Intensity (0–5)	3.2	.16	.23
Pain Interference (0–24)	15.5	1.34	2.04
Quality of Life QoL (0–100)	41.0	4.61	6.49
Depressive Symptoms PHQ-8 (0–24)	7.9	.98	1.22

## Discussion

To our knowledge, this is the first RCT assessing the efficacy of at-home tDCS for chronic pain in patients with ESKD/HD. tDCS is a cutting-edge, nonpharmacological analgesic approach, and at-home tDCS is a new approach that may facilitate long-term treatment of chronic conditions and overcome the limitations of previous short-term research-center-based tDCS interventions. Our at-home device has a remote-supervision element that also allows for enhanced outreach and better communication and interaction among patients, caregivers, and research staff.

Unlike previous studies of tDCS for pain, this trial includes a large sample, allowing meaningful evaluation of characteristics that could potentially influence outcomes. These include variation in the pain and patient characteristics, such as race/ethnicity. The study evaluates a longer treatment period than most tDCS studies and assesses both short-term and longer-term outcomes.

The unique features of this trial will enhance the understanding of short- and long-term analgesic effects of tDCS and determine whether treatment of chronic pain in an ESKD/HD population is affected by race/ethnicity or other patient characteristics. At-home tDCS is a promising nonpharmacologic treatment for pain in ESKD/HD. Establishing its long-term effects could transform the way pain is managed in this ethnically diverse growing population of patients.

## Data Availability

The original contributions presented in the study are included in the article/Supplementary Material, further inquiries can be directed to the corresponding authors.
